# Impact of a Transition from Respiratory Virus Shell Vial to Multiplex PCR on Clinical Outcomes and Cost in Hospitalized Children

**DOI:** 10.3390/children4010003

**Published:** 2017-01-07

**Authors:** Pui-Ying Iroh Tam, Lei Zhang, Zohara Cohen

**Affiliations:** 1 Division of Pediatric Infectious Diseases and Immunology, University of Minnesota Masonic Children’s Hospital, Minneapolis, MN 55454, USA; 2 Malawi-Liverpool Wellcome Trust, Blantyre 3, Malawi; 3 Clinical and Translational Science Institute, University of Minnesota, Minneapolis, MN 55454, USA; zhangl@umn.edu (L.Z.); zacohen@umn.edu (Z.C.)

**Keywords:** respiratory virus, multiplex PCR, shell vial, cell culture, diagnostics, children

## Abstract

While respiratory virus PCR panel (RVPP) is more expensive than shell vial (SV) cell culture, it has been shown to reduce unnecessary diagnostic procedures, decrease the inappropriate use of antimicrobials, and shorten the hospital length of stay (LOS). We therefore hypothesized that, for hospitalized children, RVPP would be associated with improved clinical outcomes but higher hospital charges than SV cell culture. We performed a retrospective cohort study of hospitalized children. Multivariate analysis was performed, and *p*-values were calculated. Respiratory virus testing was collected in a total of 1625 inpatient encounters, of which 156 were tested positive by RVPP (57.7%) and 112 were tested positive by SV (11.1%, *p* < 0.05). Excluding human rhinovirus (HRV) and human metapneumovirus (hMPV) from the analysis, patients with a positive test from SV had more comorbidities (*p* = 0.04) and higher mortality (*p* = 0.008). Patients with a positive test from RVPP had shorter LOS (*p* = 0.0503). Hospital charges for patients with a positive test from RVPP were lower, but not significantly so. When a multivariate analysis was performed, there were no statistically significant differences in comorbidities, mortality, LOS, or median hospital charges between those patients with a positive SV and those with a positive RVPP. Although testing with RVPP significantly increased the detection of respiratory viruses, clinical outcomes remained comparable to those tested with SV, however RVPP was found to not be associated with higher long-term hospital costs.

## 1. Introduction

Viral respiratory tract infections result in significant morbidity in children [1,2,3,4,5]. Additionally, due to the difficulty in clinically distinguishing between bacterial and viral infections, delays in diagnosis can lead to delayed antiviral therapy, inappropriate antibacterial use, and inadequate infection control cohorting. The gold standard for the identification of respiratory viruses has been cell culture. However, not all viruses are cultivable, and for those that are, it can be labor-intensive and take days to obtain a result [6]. The innovation of shell vial (SV) cell culture has allowed for reasonable turnaround times for even slow-growing clinically important human viruses [7,8].

The emergence of more rapid and sensitive molecular tests, such as a respiratory virus PCR panel (RVPP) has led to even more timely treatment, reduced unnecessary diagnostic procedures, decreased inappropriate use of antimicrobials, and shortened hospital length of stay (LOS) [9,10,11,12,13]. However, RVPP requires substantial fixed costs to purchase equipment and is more expensive in the short-term ($127 per test) compared with SV ($46 per test). It is unclear whether the use of more sensitive molecular diagnostics (compared with conventional culture methods) is associated with comparably improved clinical outcomes for hospitalized children. We hypothesized that patients with a positive RVPP test, compared with a positive SV test, would be associated with improved clinical outcomes for hospitalized children, but involve higher hospital charges.

## 2. Materials and Methods

This was a retrospective cohort study utilizing data from the Fairview Health System of Minnesota, which comprises four community hospitals with pediatric units, and the University of Minnesota Masonic Children’s Hospital, a 96-bed academic tertiary care children’s hospital. Children up to 18 years of age who received respiratory viral testing in the emergency department or in an inpatient unit between January 2011 and April 2015 were included. Readmissions within a 2-month interval were excluded, while readmissions ≥2 month intervals were treated as independent events, therefore some patients had more than one test included within the analysis. Patients discharged from the emergency department were excluded, as we could not track their clinical outcomes.

### 2.1. Data Extraction

All data were obtained from the patient`s medical record. These included clinical diagnoses that were based on International Classification of Diseases, Ninth Revision (ICD-9) codes for viral pneumonia, bacterial pneumonia, bacteremia, bacterial meningitis, and urinary tract infection. Comorbid diagnoses were also identified using ICD-9 codes for asthma, cystic fibrosis, other pulmonary, cardiovascular, metabolic including diabetes mellitus, hepatic, renal, hematologic, immunosuppression, and neurologic conditions. Other potential comorbidities considered as risk factors for influenza were also examined, including pregnancy, morbid obesity, and long-term use of aspirin. In addition, we documented need for antimicrobials, ventilator management, including continuous positive airway pressure, ICU admission, LOS, readmission, and mortality. Hospital charges were matched to the patient encounter and obtained from Strata (http://www.stratadecision.com; Strata Decision Technology, Chicago, IL, USA). This study was approved by the Institutional Review Board of the University of Minnesota.

### 2.2. Respiratory Viral Testing

Nasal and throat swabs were collected from patients, placed in transport media, and refrigerated until they were processed within 24 h of collection in the Infectious Diseases Diagnostic Laboratory at the University of Minnesota Medical Center, Fairview. At our central clinical diagnostic laboratory, RVPP was introduced in January 2014 and SV was discontinued in May 2015.

For SV testing, R-Mix SVs (Diagnostic Hybrids, Athens, OH, USA) were handled as per manufacturer’s instructions. SVs were activated by incubation at 37 °C for 2–4 h prior to inoculation. Growth medium was substituted with Refeed Medium (Diagnostic Hybrids) immediately prior to infection, after which they were inoculated with 0.2 mL of the clinical specimen. Thereafter, R-Mix SVs were centrifuged as above and incubated at 37 °C. SV monolayers were scraped after 24, 48, and 120 h of incubation at 37 °C; spotted onto slides; fixed with acetone; stained with specific monoclonal antibodies for influenza A and B, parainfluenza types 1–3, respiratory syncytial virus (RSV), and adenovirus; and read under fluorescence microscopy. A positive result was defined as the presence of bright green fluorescence in the cytoplasm of ≥2 cells. A negative result was defined as <2 fluorescent cells independent of the number of cells present on the slide.

For RVPP testing, the assay (GenMark’s eSensor Respiratory Viral Panel, GenMarkDx, Carlsbad, CA, USA) incorporates a multiplex reverse transcription-PCR amplification followed by suspension array detection, covering adenovirus groups B, C, and E; influenza A virus (including subtype determination); influenza B virus; human metapneumovirus (hMPV); parainfluenza virus types 1, 2, and 3; respiratory syncytial virus (RSV) types A and B; and human rhinovirus (HRV) [14]. The test was performed daily Monday to Friday, with results typically available in 24 h.

Only the first positive respiratory viral test performed during the hospital inpatient stay was included. The following were excluded from the analysis: (1) any tests (whether SV or RVPP) following the first positive test; and (2) tests that were only positive for HRV and hMPV, given that these were available on RVPP but not SV.

### 2.3. Statistical Methods

Generalized estimating equations were used to calculate *p*-values, to account for within-subject correlation (i.e., patients with multiple hospitalizations), in the form of linear regression, logistic regression, and multinomial regression, depending on the outcome variable. Multivariate analysis was performed controlling for age at admission, sex, race, and a number of comorbidities. Main outcomes examined were need for mechanical ventilation, LOS, readmission, and mortality. Secondary outcome measures were antimicrobial usage and hospital charges. Due to skewed distribution, LOS and hospital charge were transformed to their natural logs. Chi-square test was performed to compare cumulative probabilities. All *p*-values are two-sided and ≤0.05 was considered statistically significant. Data management was performed using Microsoft Excel 2011 (Version 14.5.6), and statistical analyses were performed using SAS (Version 9.3; SAS Institute, Cary, NC, USA).

## 3. Results

A total of 1625 hospital encounters with respiratory virus testing were collected during the study period, of which 1012 patients were tested with SV, and 613 patients with RVPP. Of these, a total of 466 tests were positive for a respiratory virus infection, from 436 patients. 112/1012 patients (11.1%) were tested positive with SV, and 354/613 patients (57.7%) were tested positive with RVPP (Table 1).

The most common respiratory viruses identified using RVPP were HRV (55.7%), followed by RSV (21.8%) and hMPV (11.3%). The most common respiratory viruses identified by SV were RSV (55.4%), followed by adenovirus (10.7%) and parainfluenza type 1 (9.8%). Ten patients had viral coinfection, most commonly including HRV (70%) and hMPV (30%), and one patient had three respiratory viruses detected on one RVPP, consisting of HRV, hMPV, and adenovirus. The only statistically significant difference noted between patient groups was the larger number of positive diagnoses for HRV and hMPV by RVPP tests among children 2–23 months and 6–10 years of age.

Excluding HRV and hMPV, there were 268 positive respiratory viral tests from hospital encounters of 263 patients, of which 112 were tested by SV culure (11.1%) and 156 by RVPP (25.4%, *p* < 0.05; Table 2). Although the study period spanned 2011 to early 2015 and RVPP was only introduced in our laboratory in January 2014, there were significantly more positive results for respiratory viruses identified by RVPP than by SV (*p* < 0.0001). With the exclusion of the additional respiratory viruses detected on RVPP (HRV and hMPV) but not SV, univariate analysis revealed that patients with a positive SV had a significantly higher number of comorbidities (2.7 versus 2.2, respectively, *p* = 0.04) and significantly higher mortality (9% versus 1%, respectively, *p* = 0.008; Table 2). Patients with positive RVPP had shorter LOS, which approached statistical significance (*p* = 0.0503). Median hospital charges for patients with a positive RVPP were lower, but not significantly so.

After controlling for potential confounders in multivariate analysis, there were no statistically significant differences in mortality, comorbidities, LOS, or median hospital charges between those patients with a positive SV compared to those with a positive RVPP (Table 3). When we compared the full panel of RVPP respiratory virus detection (i.e., including HRV and hMPV) with SV, our results remained the same, with no statistically significant differences noted. In addition, subgroup analysis was performed by age group (2–23 months, 2–5 years, 6–18 years), and no significant associations were identified.

## 4. Discussion

This study showed that a transition to testing with a multiplex PCR led to significantly increased detection of respiratory viruses, along with comparable clinical outcomes and no increase in hospital charges compared to conventional SV. Thus, our hypothesis that RVPP testing, when compared with SV testing, would be associated with improved clinical outcomes for hospitalized children but involve higher hospital charges, was refuted. While it is already well-known that molecular techniques lead to increased detection of respiratory viruses, the higher likelihood of identification of a respiratory virus may have contributed to de-escalation of antibiotics and reduced ancillary testing, thereby offsetting the cost of the more expensive PCR test. These findings are also consistent with other studies that demonstrate respiratory multiplex PCR to be a cost-effective strategy [15].

Advances in molecular technology have permitted the sensitive and rapid detection of respiratory viruses in clinical specimens [16], as timely results are considered important in affecting the clinical decisions that are made [17]. RVPP is more sensitive than SV [18] and other conventional diagnostic tests [19], and this was apparent in our study where HRV and hMPV detection accounted for two-thirds of positive RVPP results. In order to strictly compare only the respiratory viruses present in both SV and RVPP, we did not include HRV and hMPV in the above analysis so as to avoid skewing the analysis based on the clinical characteristics and outcomes of those respiratory viruses. However, even with the inclusion of these additional viruses, our results remained the same. This reflects the primary benefit of RVPP diagnostics, which is the ability to detect a broader array of respiratory viruses. Despite this, our results reflect other study findings, where increased diagnostic yield of molecular detection does not necessarily lead to a concurrent decrease in antimicrobial use [20], nor shorter LOS [21,22].

A study among immunocompromised pediatric patients—where the overall sensitivity of SV was documented at 97%—considered that management based on the negative predictive value of SV would have saved their patients cumulatively months of unnecessary isolation [23]. A study that retrospectively evaluated 202 children hospitalized with community-acquired pneumonia found that the use of RVPP was associated with a more complicated clinical course, and that detection of a respiratory virus did not influence antibiotic therapy [24]. However, RVPP was more commonly performed in patients who had severe pneumonia [24]. Another retrospective study found an association between a positive RVPP and a shorter duration of intravenous antimicrobial administration, as well as with decreased LOS and shorter duration of antimicrobials in patients with some common respiratory diagnoses [25].

Findings from prospective studies have been more mixed. One controlled clinical trial utilizing real-time PCR identified pathogens among 82% of specimens; however, rapid reporting of these results did not lead to decreases in hospital admissions, shorter LOS, or less antimicrobial use for children with acute respiratory infections [22]. In contrast, another randomized prospective controlled trial using rapid influenza diagnostics in the emergency department showed that physician awareness of a rapid diagnosis result significantly reduced the number of laboratory tests and radiographs ordered and their associated charges, decreased antibacterial use, increased antiviral use, and decreased length of time to discharge [10].

Limitations of our study pertain to the constraints of the retrospective design. This was a multicenter study that included a tertiary children’s hospital, where RVPP was recommended for patients who are immunocompromised; thus, findings may not be generalizable to other settings. In addition, all patients who had positive respiratory viral testing were included, regardless of their clinical diagnosis for that hospitalization and regardless of their underlying comorbidities. We controlled for both of these potential confounders in our multivariate analysis. However, we did not control for hospital site, which may reflect local differences in practice style, nor did we control for medical insurance status; stricter cohorting of our population may have aided in revealing additional associations. We did not look specifically at the turnaround time in assessing the impact of testing on clinical outcome.

In addition, as most of the SV specimens were obtained in the years prior to the introduction of RVPP, differences in antimicrobial usage and outcomes correlating with the type of respiratory viral test could be explained by changes in practice over time. The higher proportion of RSV detected on SV compared to RVPP (55.4% vs. 21.8%) can be attributed to the limited array of respiratory viruses tested on SV. When we performed multivariate analysis of all patients who had respiratory viral testing performed during the study period, mortality and LOS were significantly lower among those who had RVPP testing (*p* < 0.0001, *p* = 0.0008, and *p* < 0.0001, respectively) compared to those with SV testing. Hence, observed differences may reflect overall differences in the patients that were selected for respiratory viral testing during their respective time periods.

Few studies have compared the impact of different diagnostic methods on clinical outcomes and hospital costs. Our results suggest a role for respiratory viral multiplex PCR in hospitalized pediatric patients which—although more expensive—increases the detection of respiratory viruses, and is associated with clinical outcomes comparable to SV testing without an associated increase in hospital costs.

## Figures and Tables

**Table 1:** Characteristics of the total study populations who tested positive using respiratory virus PCR panel (RVPP) or shell vial (SV) cell culture.

	RVPP (*n* = 354)	SV (*n* = 112)	*p*-Value
**Age (%)**			0.015
2–23 months	187 (52.8)	74 (66.1)	
2–5 years	102 (28.8)	24 (21.4)	
6–10 years	30 (8.5)	5 (4.5)	
11–18 years	35 (9.9)	9 (8.0)	
**Male sex (%)**	187 (52.8)	56 (50.0)	0.47
**Race/ethnicity (%)**			0.93
White	214 (62.8)	68 (61.2)	
Black	67 (19.7)	16 (15.1)	
Hispanic	24 (7.0)	12 (11.3)	
Other	36 (10.6)	10 (9.4)	
**Comorbidity (%)**			
Total number, mean (range)	2.44 (0–8)	2.69 (0–8)	0.14
Any comorbidity	314 (88.7)	95 (84.8)	0.32
**Respiratory virus(es) isolated (%)**			
Adenovirus	32 (9.0)	12 (10.7)	0.58
Human metapneumovirus (hMPV)	40 (11.3)	0 (0)	<0.0001
Human rhinovirus (HRV)	197 (55.7)	0 (0)	<0.0001
Influenza A	14 (4.0)	6 (5.4)	0.55
Influenza B	10 (2.8)	3 (2.7)	0.93
Parainfluenza 1	3 (0.9)	11 (9.8)	0.002
Parainfluenza 2	6 (1.7)	3 (2.7)	0.56
Parainfluenza 3	23 (6.5)	8 (7.1)	0.81
Respiratory syncytial virus (RSV)	77 (21.8)	62 (55.4)	<0.0001
>1 respiratory virus isolated	45 (12.7)	0 (0)	<0.0001

*n* = number of patients.

**Table 2:** Characteristics and comparison of the two study populations, excluding HRV and hMPV.

	RVPP (*n* = 156)	SV (*n* = 112)	*p*-Value
**Age (%)**			0.22
2–23 months	92 (59)	74 (66)	
2–5 years	39 (25)	24 (21)	
6–10 years	10 (6)	5 (4)	
11–18 years	15 (10)	9 (8)	
**Male sex (%)**	80 (51)	56 (50)	0.78
**Race/ethnicity (%)**			0.65
White	98 (65)	68 (64)	
Black	30 (20)	16 (15)	
Hispanic	10 (7)	12 (11)	
Other	12 (8)	10 (9)	
**Comorbidity (%)**			
Total number, mean (range)	2.2 (0–8)	2.7 (0–8)	0.04
Any comorbidity	137 (88)	95 (85)	0.47
**Respiratory virus(es) isolated (%)**			
Adenovirus	33 (21)	12 (11)	0.022
Influenza A	14 (9)	6 (5)	0.25
Influenza B	10 (6)	3 (3)	0.1
Parainfluenza 1	3 (2)	11 (10)	0.011
Parainfluenza 2	6 (4)	3 (3)	0.59
Parainfluenza 3	23 (15)	8 (7)	0.047
Respiratory syncytial virus	77 (49)	62 (55)	0.34
**Medications**			
Received antivirals			
Oseltamivir	16 (10)	13 (12)	0.76
Ribavirin	1 (1)	6 (5)	0.035
Received antibacterials			
Vancomycin	31 (20)	38 (34)	0.13
Ceftriaxone/Cefotaxime	26 (17)	20 (18)	0.78
Penicillin/Ampicillin	2 (1)	1 (1)	0.76
**Clinical course and management**			
Required mechanical ventilation (%)	69 (44)	44 (39)	0.42
Median length of hospital stay (LOS) *, (range)	3.6 (0.1–133.0)	3.9 (0.7–304.3)	0.05
LOS in log scale, mean (*SD*)	1.40 (0.98)	1.65 (1.23)	0.062
Readmission within 14 days (%)	7 (4)	7 (6)	0.53
Mortality (%)	2 (1)	10 (9)	0.008
**Hospital charges**			
Median, dollars (range)	33,796 (2953–4,015,593)	27,373 (1099–4,068,499)	
Charges in log scale, mean (*SD*)	10.57 (1.16)	10.74 (1.58)	0.29

*SD*: standard deviation; *, days.

**Table 3:** Multivariate analysis evaluating clinical course and management of children who tested positive with RVPP versus SV testing, excluding HRV and hMPV.

Outcome	OR (95% CI)	*p*-Value
Required mechanical ventilation	1.24 (0.72, 2.14)	0.43
Log LOS	−0.14 (−0.39, 0.11) *	0.27
Readmission within 14 days	1.05 (0.32, 3.44)	0.94
Mortality	0.21 (0.04, 1.16)	0.074
Antiviral usage	0.64 (0.28, 1.47)	0.29
Antibacterial usage	0.71 (0.40,1.25)	0.23
Log (hospital charges)	0.001 (−0.33, 0.33) *	0.99

OR, odds ratio; *, difference in LOS/hospital charges (not odds ratio).
